# The cabbage-leaf water extract can inhibit the germination and seedling growth of three receptor crops

**DOI:** 10.3389/fpls.2025.1609150

**Published:** 2025-08-07

**Authors:** Cong Zhao, Meihua Ye, Ting Pan, Min Zhao, Nana Li, Yueyue Xu, Xuefang Huang, Juanling Wang

**Affiliations:** ^1^ Shanxi Institute of Organic Dryland Farming, Shanxi Agricultural University/Key Laboratory of Sustainable Dryland Agriculture of Shanxi Province, Shanxi Agricultural University, Taiyuan, Shanxi, China; ^2^ Key Laboratory of Sustainable Dryland Agriculture (Co-construction by Ministry of Agriculture and Rural Affairs and Shanxi Province), Shanxi Agricultural University, Taiyuan, Shanxi, China; ^3^ National Agricultural Environment Observation and Experimental Station in Jinzhong, Shanxi Agricultural University, Taiyuan, Shanxi, China; ^4^ College of Agronomy, Shanxi Agricultural University, Taiyuan, Shanxi, China

**Keywords:** *Brassica oleracea* L. var. capitata L., water extract, allelopathic effect, malondialdehyde, antioxidant enzyme

## Abstract

Cabbage cultivation has been observed to significantly hinder the growth of subsequent crops in the cold, arid regions of Shanxi Province. To investigate the allelopathic effects of cabbage (*Brassica oleracea* L. var. *Capitata* L.) on seed germination and seedling growth of three crops commonly cultivated in such areas, we studied the effects of water extracts from air-dried cabbage leaves on cocozelle, kidney bean, and corn. Experiments were conducted using both petri dish filter paper and pot culture methods to simulate natural conditions. The results indicated that cabbage leaf water extracts at concentrations of 0.01-0.04 g·mL^-1^ significantly inhibited seed germination. At higher concentrations (0.06-0.08 g·mL^-1^), the extract markedly suppressed seedling growth in all three crops (p < 0.05), with the degree of inhibition increasing alongside concentration. Radicle elongation in cocozelle and corn was more strongly inhibited than germ elongation at the same extract concentration, In contrast, kidney bean showed greater inhibition of germ elongation than radicle elongation at 0.04 g·mL^-1^. Malondialdehyde (MDA) content was elevated in kidney bean and corn seedlings treated with cabbage extract, indicating oxidative stress. At extract concentrations of 0.06-0.08 g·mL^-1^, antioxidant enzyme activities, such as antioxidase superoxide dismutase (SOD), peroxidase (POD) and catalase (CAT) were enhanced in all three crops. The comprehensive allelopathic inhibition followed the order: cocozelle > corn > kidney bean. The differences appear to be related to the changes in MDA content and antioxidant enzyme activity. Based on these findings, rotating cabbage and kidney bean may help reduce negative allelopathic effects. It is also recommended to remove the entire aboveground portion of cabbage during harvest to reduce allelochemical residues in the soil and minimize their inhibitory impact on subsequent crops.

## 1 Introduction

Seed is fundamental to agricultural production, and its germination performance directly impacts crop success (Liu et al., 2021). Germination is a complex physiological process, not only influenced by internal factors such as hormone levels, nutrient accumulation, seed coat disorder and genetic factors, but also by environmental conditions like water availability, temperature, light, and salinity. Among these, the inhibitory effects of soil-accumulated allelochemicals are particularly noteworthy. These compounds, which are secondary metabolites released by living plants through roots, aboveground volatilization, rain and fog leaching or decomposition of plant residues, can accumulate continuously in soil and suppress the growth of surrounding vegetation (Zhang and Lin, 2009; Guo et al., 2019). Their concentrations depend on several factors, including plant biomass, litter density, decomposition rate and precipitation. Once accumulated beyond a threshold, allelochemicals can significantly inhibit both seed germination and seedling growth, thereby affecting the competitive dynamics of plant communities (Wang et al., 2012; Li et al., 2022; Fan et al., 2021).

Numerous studies have shown that medicinal plants often exhibit strong autotoxicity, and exert significant allelopathic effects on other crops (Chen et al., 2024; Gao et al., 2009). Similarly, certain vegetables, such as tomato, garlic, green onion, cucumber and amaranth also have demonstrated clear allelopathic inhibition on the germination and growth of neighboring crops (Xu et al., 2017; Zhao et al., 2019; Yao et al., 2017; You, 2007; Guan, 2015). However, there are limited researches on the allelopathic effects of cabbage (Zhao et al., 2022; Kural and Özkan, 2020).

*Brassica oleracea* L. var. *capitata* L., an annual or biennial herb of the Cruciferae family, is a cultivated variety of *Brassica oleracea* L. Modern nutritional studies have shown that cabbage is rich in bioactive compounds, particularly isothiocyanate (Tang et al., 2013) and phenolic acids (Cartea et al., 2011). Both of them possess anti-tumor (Ono et al., 2023) and antimicrobial properties (Liu et al., 2022; Meng et al., 2023). As a result, cabbage plays an important role in promoting public health (Nooyens et al., 2011). However, phenolic acids are also recognized as major allelochemicals (Rice, 1984), and isothiocyanates, hydrolysis products of glucosinolates unique to Brassica species, are also allelopathic in nature (Hanschen, 2024).

In the cold, arid regions of Shanxi Province, cabbage cultivation has been observed to significantly hinder the growth of subsequent crops, including non-cruciferous species. For example, after 45 days of sowing, corn grown following cabbage had only 60% of the plant height and two fewer leaves compared to corn grown after tomato. Additionally, purple discoloration was observed on the basal leaves, indicating stress and reduced early growth, which could negatively impact yield (Zhang et al., 2017). With the increasing scale of cabbage production, these allelopathic effects have become more pronounced, highlighting the importance of further investigation.

In this study, air-dried cabbage leaves were used as donor materials to simulate allelopathic leaching from rain and fog. We examined their effects on seed germination and seedling growth of cocozelle (*Cucurbita pepo* L.), kidney bean (*Phaseolus vulgaris* L.) and corn (*Zea mays* L.), which are commonly grown in the dryland of Shanxi province. Additionally, we analyzed MDA content and antioxidant enzyme activity in the receptor crops to investigate the physiological mechanisms underlying the observed allelopathic effects. This research aims to clarify the causes of replanting obstacles in cabbage cultivation and provide a theoretical foundation for designing rational crop rotation strategies.

## 2 Materials and methods

### 2.1 Experimental material

The donor material used in this study was the leaves of heading cabbage (*Brassica oleracea* L. var. *capitata* L. cv. ‘Iron General’), collected from the experimental base in Hecun Village, operated by the Shanxi Institute of Organic Dryland Agriculture, Shanxi Agricultural University. The region receives an average annual precipitation of 450 mm, mainly concentrated betweeen June and September, with concurrent periods of heat and rainfall.

The receptor crops were cocozelle (*Cucurbita pepo* L. cv. ‘Ataiyidai’), kidney bean (*Phaseolus vulgaris* L. cv. ‘Jinlüwang’), and corn (*Zea mays* L. cv. ‘Bingdan16’). All procured from the Shanxi Agricultural High-Tech Market.

### 2.2 Preparation of cabbage leaf water extract

Cabbage leaves were washed and air-dried indoors, then ground and sieved through a 40-mesh screen (aperture 0.42 mm). 100 g of the powdered leaves was soaked in 1000 mL of distilled water at a constant temperature (25°C) for 48 h. After standing, the solution was filtered through double-layer quantitative filter paper to obtain a mother liquor with a concentration of 100 g·L^-1^. This mother liquor was then diluted with distilled water to prepare extracts at concentrations of 0.01, 0.02, and 0.04 g·mL^-1^ for seed germination experiments, and 0.06, 0.08, 0.10 g·mL^-1^ for seedling growth studies. All extracts were stored at 4°C until use.

### 2.3 Seed germination measurements

Thirty uniform, healthy seeds of each crop (corn, cocozelle and kidney bean) were disinfected with 75% ethanol for 10 min, rinsed and placed evenly on sterile petri dishes (diameter 15 cm) lined with a double-layer filter paper. Each dish received 20 mL of cabbage-leaf water extract at concentrations of 0.01, 0.02 and 0.04 g·mL^-1^.Distilled water was used as the control. Dishes were incubated in the dark at 25°C and 60% humidity. Water lost to evaporation was replenished by weighing to maintain consistent solution concentration and moisture (Li et al., 2022).

Four replicates were prepared per treatment. The number of germinated seeds was recorded every 24 hours. Seeds were considered germinated when the radicle exceeded 50% of the seed length. After 7 days, the bud and root length were measured. Germination potential, germination rate, germination index and vigor index were calculated as follows:


Germination potential=Number of germinated seeds at peak/Totalnumber of testedseeds

Germination rate=Totalnumber of germinated seeds/Totalnumber of testedseeds

Germination index (GI)=åGt/Dt

Vigor index (VI)=GI×Sx

where Gt indicates the number of germinated seeds on day t, Dt indicates seed germination days, Sx indicates the average radicle length.

### 2.4 Seedling growth status and physiological indices

Seedling growth was assessed via pot culture. Surface soil (non-cabbage stubble) was sieved through a 20-mesh screen (pore diameter 0.850 mm). The water content of soil was adjusted to 20%. Each pot (top side: 10 cm, bottom side: 7 cm, height: 8 cm) was filled with 250 g of soil below and 200 g above the seed. Two seeds were sown per pot, and after emergence, thinned to one uniform seedling per pot. Plants were grown in an artificial climate box with 12-hour light, day, 4000 lx intensity, and temperature/relative humidity set to 25°C/50% during the day and 20°C/60% at night.

Ten replicates were set up for each treatment. At least four uniform seedlings per crop were selected for treatment. 20 mL of cabbage-leaf aqueous extracts with concentrations of 0.06, 0.08 and 0.10 g·mL^-1^ were applied every two days respectively, and the same amount of distilled water served as control. Experiment continued for 20 days under uniform management conditions.

The plant height, root length and aboveground/belowground dry weight were recorded on the last day of the treatment. Leaves were collected at 9:00 AM, wrapped in foil, and immediately frozen in liquid nitrogen. Samples were stored at -40°C for physiological analysis.

MDA content was measured using the thiobarbituric acid (TBA) method (Xu et al., 1992). SOD activity was determined via nitrotetrazolium blue (NBT) photochemical reduction. Once unit of SOD activity was defined as the enzyme amount that inhibits NBT reduction by 50% per mg protein in 1 mL of reaction solution. CAT activity was measured via UV absorption. One unit of activity corresponded to the decomposition of 1 μmol of H_2_O_2_ per mg of protein per second. POD activity was determined by guaiacol colorimetry, where one unit was defined as the enzyme amount required to produce 1 µg of substrate per minute per mg of protein at 37°C (Zou, 2001).

### 2.5 Statistical analysis

Significance testing between treatments and the control was conducted using Duncan ‘s new multiple range test in SPSS 18.0. The response index (*RI*) was used to assess the type and intensity of allelopathy.


$$RI=[\begin{array}{l} 1-C/T,\;T\geq C\\ T/C-1,\;T<C \end{array}$$

Where *C* is the control value, *T* is the treatment value. *RI* > 0 indicates a promoting effect, *RI*< 0 indicates a inhibitory effect, and the absolute value reflects the intensity of effect.

To comprehensively evaluate allelopathic effects, a composite effect index was calculated as the arithmetic mean of all *RI* values for each crop under the same treatment. This included germination potential, germination rate, shoot/root length, and shoot/root dry weight.

## 3 Results

### 3.1 Seed germination responses of the three receptor crops

**Figure 1:**
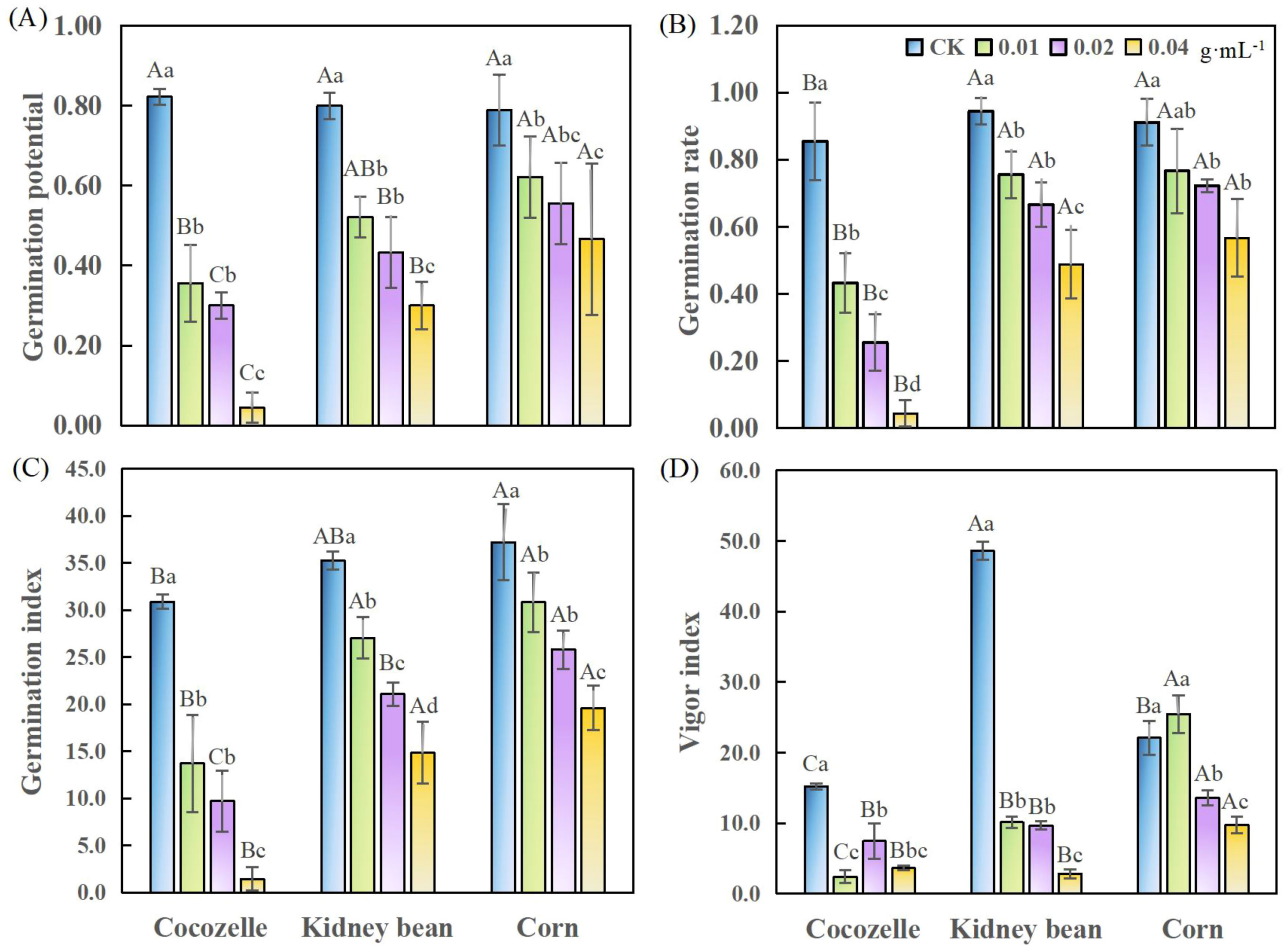
Effects of different concentrations of cabbage-leaf water extracts on the seed germination indices of three crop species. **(A)** germination potential; **(B)** germination rate; **(C)** germination index; **(D)** vigor index. Different capital letters denote significant differences among crops under the same treatment (*p*< 0.05). Different lowercase letters denote significant differences within the same crop across different concentrations (*p*< 0.05). The same below.

As the concentration of cabbage-leaf water extract increased, the germination potential, germination rate and germination index of all three tested crops decreased progressively (**Figure 1**). The extracts of all tested concentrations significantly inhibited the germination potential of the seeds (*p*< 0.05), though the degree of inhibition varied by crop (**Figure 1A**). Cocozelle was the most sensitive: at the highest extract concentration (0.04 g·mL^-1^), its germination potential dropped by 76.8% compared to the control. Even at the lowest concentration (0.01g·mL^-1^), the reduction remained significant at 56.9% (*p*< 0.05). For kidney beam germination potential decreased by 34.7% and 62.5% at 0.01 g·mL^-1^ to 0.04 g·mL^-1^, respectively. Corn was less sensitive overall, with its germination potential reduced by 21.2% at the lowest concentration and by 40.8% at the highest (p< 0.05). These results highlight a clear dose-response relationship: increasing extract concentration leads to progressively stronger inhibition of germination potential across all three crops. When treatment concentration exceeded 0.02 g·mL^-1^, the differences between crop responses became more pronounced (p< 0.05).

All tested concentrations of the extract significantly reduced the germination rates of cocozelle and kidney bean (*p*< 0.05). In contrast, corn showed a significant reduction only at concentrations ≧0.02 g·mL^-1^. As shown in **Figure 1B**, cocozelle again displayed the strongest response. At 0.04 g·mL^-1^, its germination rate decreased by 95%, and even at the lowest concentration (0.01 g·mL^-1^), the inhibition reached 49%.

The germination index also declined with rising extract concentration, following a trend similar to that of germination potential and rate (**Figure 1C**). Cabbage-leaf extract substantially reduced cocozelle’s germination index relative to other crops. In the absence of treatment, kidney bean exhibited the highest vigor index, followed by corn and cocozelle. After extract application, corn’s vigor index became significantly higher than the others (*p*< 0.05). All concentrations significantly lowered the vigor index of cocozelle and kidney bean (*p*< 0.05), while corn showed significant reduction only at concentrations ≧ 0.02 g·mL^-1^. Notably, at 0.01 g·mL^-1^, cocozelle’s seed vigor was reduced by 84%, the most among the crops. For kidney bean and corn, the greatest reductions occurred at 0.04 g·mL^-1^, with vigor declining by 94% and 56%, respectively. Interestingly, at the lowest concentration, corn’s vigor index increased by 15% compared to the control (**Figure 1D**).

**Figure 2:**
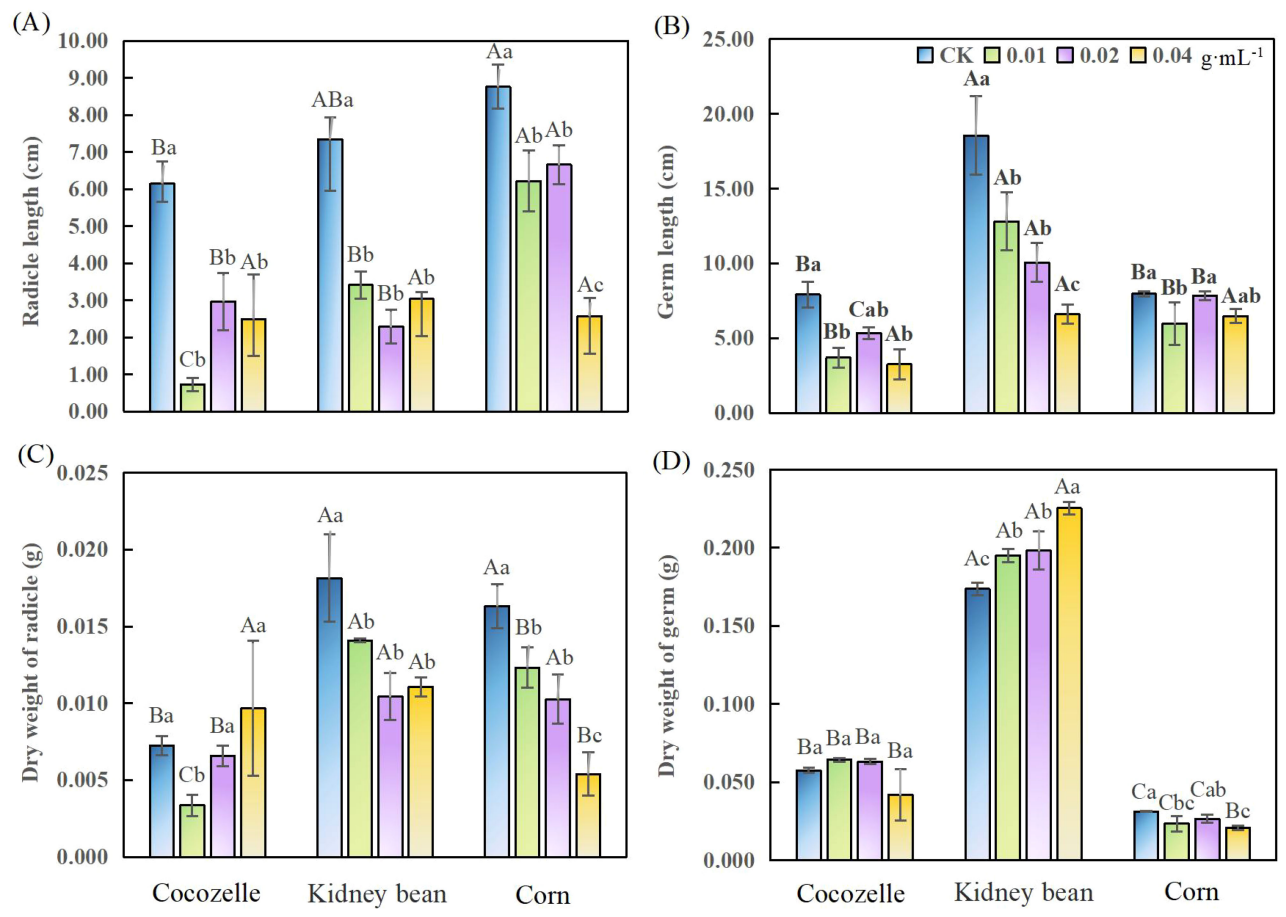
Effects of cabbage-leaf water extracts on radicle and germ growth and dry weight of three crop seedlings. **(A)** radicle length; **(B)** germ length; **(C)** radicle dry weight; **(D)** germ dry weight. Different lowercase letters denote significant differences within the same crop across different concentrations (p< 0.05).

Cabbage-leaf water extract significantly inhibited radicle elongation in all three crops (*p<* 0.05), but the concentration at which inhibition peaked occurred at different levels for each crop (**Figure 2A**). For cocozelle, radicle length decreased most at the lowest concentration (0.01 g·mL^-1^), with an 88% reduction. Kidney bean radicle length was most affected at the intermediate concentration (0.02 g·mL^-1^), decreasing by 69%. Corn showed the greatest reduction, about 71%, at the highest concentration (0.04 g·mL^-1^).

Compared with the control, cabbage-leaf extract significantly reduced kidney bean germ length at all concentrations (*p*< 0.05). The germ lengths of cocozelle and kidney bean were the shortest at the highest concentration (0.04 g·mL^-1^), reduced by 59% and 64%, respectively. For corn, germ length decreased most (by 25%) at the lowest concentration (0.01 g·mL^-1^), suggesting that germ elongation in corn was less sensitive to the extract (**Figure 2B**).

Radicle dry weight showed two distinct patterns across crops (**Figure 2C**) In cocozelle, dry weight initially decreased and then increased with concentration. Only at 0.01 g·mL^-1^ was the reduction significant (*p*< 0.05). For kidney bean and corn, radicle dry weight declined steadily with increasing concentration. Kidney bean showed a 43% reduction at 0.02 g·mL^-1^, and corn declined by 67% at 0.04 g·mL^-1^. Notably, although cocozelle’s radicle length was significantly reduced at 0.04 g·mL^-1^ (**Figure 2A**), its radical dry weight slightly increased, suggesting a potential thickening effect of the extract (**Figure 2C**).

Germ dry weight responses also varied by crop and concentration (**Figure 2D**). Cocozelle was largely unaffected across all concentrations. In contrast, kidney bean germ dry weight increased significantly with increasing extract concentration (*p*< 0.05), rising by 12% at 0.01g·mL^-1^ and nearly 30% at 0.04 g·mL^-1^. Corn responded differently, with dry weight significantly reduced at both 0.01 g·mL^-1^ and 0.04 g·mL^-1^ by 24% and 34%, respectively. Overall, cocozelle’s biomass appeared less responsive, while kidney bean exhibited a trade-off: radicle inhibition coupled with increased germ weight (**Figures 2C, D**).

Radicle and germ responses varied even within the same crop at a given concentration. In general, radicle length was more sensitive than germ length, except for kidney bean at 0.04 g·mL^-1^. For instance, at concentrations of 0.01, 0.02, 0.04 g·mL^-1^, corn’s radicle length was 71.04%, 76.02%, and 29.34%, while relative germ length was 75.19%, 98.41%, and 81.36%, respectively. A similar trend was observed for dry weight, radicals were typically more inhibited than germ tissues, except in cocozelle at 0.04 g·mL^-1^ and corn at 0.01 g·mL^-1^ (**Table 1**).

**Table 1:** Effects of cabbage-leaf water extract concentrations on relative radicle and germ growth of three crops.

Receptor crops	Mass concentration (g·mL^-1^)	Relative radicle length (%)	Relative germ length (%)	Relative radicle dry weight (%)	Relative germ dry weight (%)
Cocozelle	0.01	12.00 ± 1.11b	46.85 ± 2.57b	47.92 ± 8.57c	112.91 ± 1.51a
	0.02	48.35 ± 2.96a	67.51 ± 3.96a	93.85 ± 5.15b	110.58 ± 2.97a
	0.04	40.67 ± 2.15a	60.09 ± 10.22ab	138.10 ± 7.14a	73.39 ± 8.95b
Kidney bean	0.01	46.48 ± 5.28a	69.06 ± 12.55a	78.23 ± 10.23a	112.08 ± 4.88b
	0.02	31.21 ± 3.14b	54.15 ± 10.67a	57.97 ± 14.16a	113.95 ± 2.96b
	0.04	41.40 ± 2.62a	35.56 ± 3.1b	61.47 ± 9.86a	129.53 ± 3.53a
Corn	0.01	71.04 ± 9.56a	75.19 ± 10.48b	77.04 ± 11.05a	75.61 ± 12.41a
	0.02	76.02 ± 5.21a	98.41 ± 5.01a	64.14 ± 9.87a	85.58 ± 4.33a
	0.04	29.34 ± 3.58b	81.36 ± 4.39b	33.67 ± 7.24b	66.87 ± 2.15a

Different lowercase letters in the table mean the significant differences at 0.05 level between concentration treatment for the same crop. The same below.

The extract generally exerted negative allelopathic effects on all germination parameters (**Table 2**). Cocozelle showed the strongest response in germination rate at 0.04 g·mL^-1^ (allelopathic index: -0.946). Interestingly, the dry weight of cocozelle germ was least affected at 0.02 g·mL^-1^, with a weak positive index of 0.088. In some cases, cocozelle showed positive allelopathic indices for radicle and germ dry weight, indicating that specific concentrations may slightly promote growth. For kidney beans, only germ dry weight showed consistently positive allelopathic indices, while all other indicators were negatively affected. The most sensitive parameter was radicle length at 0.02 g·mL^-1^ (index: -0.688), while the least sensitive was germ dry weight at 0.01 g·mL^-1^ (index: 0.109). Corn exhibited negative allelopathic indices across all parameters and concentrations. Radicle length was most sensitive to 0.04 g·mL^-1^ (index: -0.707), while germ length responded least at 0.02 g·mL^-1^ (index: -0.016). Under the same concentration, radicle inhibition was generally stronger than germ inhibition, especially for cocozelle and corn (**Table 2**).

**Table 2:** Allelopathic indices of cabbage-leaf water extract concentrations on seed germination parameters of three crops.

Receptor crops	Mass concentration (g·mL^-1^)	Index of allelopathic effect					
		Germination potential	Germination rate	Radicle length	Germ length	Dry weight of germ	Dry weight of radicle
Cocozelle	0.01	-0.568Ba	-0.494Ba	-0.880 Ca	-0.532Aa	0.107Aa	-0.537Ba
	0.02	-0.635Ba	-0.701Bb	-0.516Ba	-0.325Ba	0.088Aa	-0.103Aa
	0.04	-0.946Bb	-0.948Bc	-0.593Aa	-0.589Ba	-0.272Ba	0.250Aa
Kidney bean	0.01	-0.347ABa	-0.200Aa	-0.535Ba	-0.309Aa	0.109Ab	-0.224Aa
	0.02	-0.458ABa	-0.294Aa	-0.688Ba	-0.458Bab	0.124Ab	-0.425Ba
	0.04	-0.625Aa	-0.482Ab	-0.586Aa	-0.644Bb	0.229Aa	-0.390Ba
Corn	0.01	-0.211Aa	-0.159Aa	-0.290Aa	-0.248Aa	-0.249Ba	-0.245Aa
	0.02	-0.296Aa	-0.207Aa	-0.240Aa	-0.016Aa	-0.150Ba	-0.371Ba
	0.04	-0.408Aa	-0.378Aa	-0.707Ab	-0.187Aa	-0.336Ba	-0.670Bb

Different lowercase letters denote significant differences within the same crop across different concentrations (p < 0.05).

### 3.2 Seedling growth responses of the three receptor crops

Seedling height and root length of all three crops decreased with increasing the concentration of cabbage-leaf water extract. At 0.08 g·mL^-1^, cocozelle seedlings height was significantly reduced compared to the control (*p<* 0.05). For kidney bean, significant reductions in both plant height and root length occurred at 0.10 g·mL^-1^ (*p<* 0.05). Similarly, 0.06 g·mL^-1^ of extract significantly reduced plant height and root length in corn seedlings (*p<* 0.05). Among the crops treated with the highest concentration, corn exhibited the most substantial decrease in seedling height and root length, by 42.3% and 57.7%, respectively, compared to the control. In contrast, cocozelle showed the least reduction, with plant height and root length decreasing by 35.6% and 47.9%, respectively (**Table 3**).

**Table 3:** Effects of mass concentrations of cabbage-leaf water extracts on the seedling growth of three crops.

Receptor crops	Mass concentration (g·mL^-1^)	Plant height		Root length		Underground dry weight		Aboveground dry weight	
		Measured value (cm)	Response index (*RI*)	Measured value (cm)	Response index (*RI*)	Measured value (g·plant^-1^)	Response index (*RI*)	Measured value (g·plant^-1^)	Response index (*RI*)
Cocozelle	CK	12.78 ± 1.65a	–	9.78 ± 0.76a	–	0.059 ± 0.009a	–	0.163 ± 0.006a	–
	0.06	9.89 ± 0.86ab	-0.226	8.28 ± 0.81ab	-0.153	0.043 ± 0.005b	-0.271	0.149 ± 0.008ab	-0.086
	0.08	9.31 ± 0.52b	-0.272	6.53 ± 0.92ab	-0.332	0.040 ± 0.009b	-0.322	0.139 ± 0.009b	-0.147
	0.10	8.23 ± 0.91b	-0.356	5.10 ± 0.55b	-0.479	0.028 ± 0.006c	-0.525	0.094 ± 0.005c	-0.423
Kidney bean	CK	25.12 ± 0.86a	–	11.98 ± 0.85a	–	0.116 ± 0.006a	–	0.289 ± 0.012a	–
	0.06	21.30 ± 1.17a	-0.152	10.52 ± 1.23a	-0.122	0.083 ± 0.009b	-0.285	0.215 ± 0.043a	-0.256
	0.08	19.85 ± 2.25ab	-0.210	8.75 ± 1.86ab	-0.270	0.078 ± 0.075b	-0.328	0.186 ± 0.027b	-0.356
	0.10	14.59 ± 1.32b	-0.419	6.16 ± 0.59b	-0.486	0.056 ± 0.007c	-0.517	0.162 ± 0.038b	-0.439
Corn	CK	46.69 ± 3.31a	–	17.80 ± 1.90 a	–	0.131 ± 0.007a	–	0.355 ± 0.005a	–
	0.06	32.27 ± 1.47b	-0.309	9.30 ± 1.24b	-0.478	0.109 ± 0.005b	-0.168	0.186 ± 0.009b	-0.476
	0.08	31.63 ± 2.27b	-0.323	9.10 ± 0.95b	-0.489	0.095 ± 0.009c	-0.275	0.228 ± 0.011b	-0.358
	0.10	26.92 ± 1.97c	-0.423	7.50 ± 0.80c	-0.579	0.074 ± 0.005c	-0.435	0.157 ± 0.004b	-0.558

Different lowercase letters in the table mean the significant differences at 0.05 level between concentration treatment for the same crop.

The dry weights of both the aboveground and underground parts of the seedlings also declined as cabbage-leaf extract concentration increased. For cocozelle, root dry weight was significantly inhibited across all concentrations (*p*< 0.05), decreasing by 27.1%-52.5% compared to the control. Aboveground dry weight was significantly reduced at 0.08 g·mL^-1^ and 0.10 g·mL^-1^ (by 15.0% and 42.2%, respectively), whereas the lowest concentration (0.06 g·mL^-1^) caused only an 8.6% reduction (**Table 3**).

Kidney bean root dry weight dropped by 28.4%-51.7% across treatments, and aboveground dry weight also showed significant decreases of 35.6% and 43.9% at 0.08 g·mL^-1^ and 0.10 g·mL^-1^, respectively (*p*< 0.05). For corn, all tested concentrations significantly reduced both root and shoot biomass (*p<* 0.05). The most severe inhibition occurred at 0.10 g·mL^-1^, with root and shoot dry weights decreasing by 43.5% and 55.7%, respectively. At 0.08 g·mL^-1^, inhibition of shoot biomass was notably reduced (*p<* 0.05), but the effect on root dry weight remained unchanged. Interestingly, at 0.06 g·mL^-1^, shoot biomass inhibition intensified (*p<* 0.05), while inhibition of root biomass was significantly alleviated, with a reduction of only 17.2% (*p<* 0.05) (**Table 3**; **Supplementary Figure S1**).

The allelopathic effect indices for cabbage-leaf extract on seedling growth indicators were negative for all three crops, indicating an overall inhibitory effect. With the exception of corn shoot dry weight, the level of inhibition generally increased with extract concentration. The strongest inhibitory effect on underground dry weight occurred in cocozelle and kidney bean at 0.10 g·mL^-1^, with allelopathic indices of -0.525 and -0.517, respectively. The weakest effects were observed in cocozelle underground dry weight and kidney bean root length at 0.06 g·mL^-1^, with indices of -0.086 and -0.122, respectively.

Overall, cabbage-leaf extract exerted stronger inhibitory effects on corn seedling height, root length, and shoot dry weight compared to the other two crops, though its effect on corn root dry weight was slightly less severe. Corn root length showed the strongest sensitivity to the highest extract concentration, with an allelopathic effect index of -0.579, while its root dry weight was least affected at 0.06 g·mL^-1^ (index: -0.168) (**Table 3**).

### 3.3 Comprehensive allelopathic effects of cabbage-leaf extract on three receptor crops

The overall allelopathic effects of cabbage-leaf water extracts on seed germination and seedling growth in cocozelle, kidney bean, and corn were consistently inhibitory across all tested concentrations. During the germination stage, the strongest inhibition occurred at the highest concentration (0.04 g·mL^-1^), with comprehensive allelopathic indices of -0.516 for cocozelle, -0.416 for kidney bean, and -0.448 for corn. As the extract concentration decreased, the inhibitory effect on kidney bean weakened gradually. For cocozelle and corn, the inhibitory effect first weakened and then intensified again.

At lower concentrations (0.01 g·mL^-1^ and 0.02 g·mL^-1^), corn showed relatively larger allelopathic index values, suggesting weaker inhibition compared to the other two crops. At 0.04 g·mL^-1^, kidney bean had the largest index (i.e., the weakest inhibition), while cocozelle consistently displayed the strongest inhibition at all concentrations during the seed germination stage.

A similar trend was observed in seedling growth. The highest concentration (0.10 g·mL^-1^) resulted in the strongest comprehensive inhibitory effects, with allelopathic indices -0.447 for cocozelle, -0.465 for kidney bean and -0.497 for corn. However, unlike in the germination stage, cocozelle was least affected during seedling growth, while corn was most affected.

Taking both seed germination and seedling growth stages into account, the overall order of allelopathic sensitivity to cabbage-leaf water extract was cocozelle > corn > kidney bean (**Table 4**).

**Table 4:** Allelopathic comprehensive effects of mass concentrations of cabbage-leaf water extracts on the seed germination and seedling growth of three crops.

Receptor crops	Concentrations of cabbage-leaf water extracts (g·mL^-1^)						Average	Ranking
	0.01	0.02	0.04	0.06	0.08	0.10		
Cocozelle	-0.484	-0.366	-0.516	-0.185	-0.267	-0.447	-0.387	1
Kidney bean	-0.251	-0.367	-0.416	-0.204	-0.291	-0.465	-0.332	3
Corn	-0.234	-0.213	-0.448	-0.356	-0.358	-0.497	-0.351	2

### 3.4 Differences in physiological characteristics of seedlings of three crops

Except for cocozelle seedlings at 0.06 g·mL^-1^, all treatments resulted in higher MDA content compared to the control. Among the three crops, only corn seedlings showed a consistent increase in MDA levels with rising extract concentrations. At the highest concentration (0.10 g·mL^-1)^, MDA content in both cocozelle and corn seedlings was significantly higher than the control (*p<* 0.05), with levels doubling relative to untreated plants. In contrast, MDA content in kidney bean seedlings remained statistically unchanged across all tested concentrations. These findings suggest that among the three crops, only kidney bean seedlings were able to maintain relatively stable membrane integrity under cabbage-leaf extract stress (**Table 5**).

**Table 5:** Effect of different concentrations of cabbage-leaf water extracts on the seedling physiological indicators from three crops.

Receptor crop	Mass concentration (g·mL^-1^)	MDA content (µmol·g^-1^)	Antioxidant enzyme activity (U·g^-1^·min^-1^ FW)		
			SOD	POD	CAT
Cocozelle	CK	3.92 ± 0.16b	51.2 ± 1.9a	20.1 ± 2.6 b	1.6 ± 0.2c
	0.06	3.78 ± 0.49b	53.8 ± 3.1b	28.5 ± 4.1 ab	5.3 ± 0.8a
	0.08	5.06 ± 1.84 a	65.4 ± 2.6a	32.8 ± 3.9a	3.9 ± 1.6b
	0.10	7.89 ± 0.61a	39.5 ± 4.8c	14.2 ± 5.4c	3.4 ± 1.1b
Kidney bean	CK	2.56 ± 0.11a	146.2 ± 10.8c	2085 ± 105b	428.7 ± 20a
	0.06	2.79 ± 0.08a	149.4 ± 9.9b	2118 ± 158b	439.1 ± 11a
	0.08	3.16 ± 0.12a	171.9 ± 5.1b	2104 ± 102b	465.5 ± 6a
	0.10	3.65 ± 0.06a	143.6 ± 4.2a	2499 ± 162a	450.6 ± 13a
Corn	CK	2.96 ± 0.28b	22.8 ± 1.3b	52.8 ± 6.4b	70.2 ± 2.7b
	0.06	3.37 ± 1.05b	34.6 ± 4.1a	63.9 ± 5.2a	72.5 ± 3.5b
	0.08	4.96 ± 2.29a	28.2 ± 3.2ab	56.7 ± 2.8b	89.3 ± 6.7a
	0.10	6.09 ± 1.18a	20.1 ± 2.4b	41.3 ± 3.5c	52.5 ± 7.4c

Different lowercase letters in the table mean the significant differences at 0.05 level between concentration treatment for the same crop.

SOD and POD activities in cocozelle seedlings initially increased with extract concentration and then declined. The most pronounced increase in both enzymes occurred at 0.08 g·mL^-1^ (*p<* 0.05), while a significant reduction was observed at 0.10 g·mL^-1^ (*p<* 0.05). In kidney bean seedlings, SOD activity peaked at 0.08 g·mL^-1^ (*p<* 0.05), while POD activity continued to rise and reached its highest level at 0.10 g·mL^-1^ (*p<* 0.05). Although corn seedlings showed a similar trend to cocozelle, the concentrations associated with peak enzyme activity differed. For corn, the highest SOD and POD activities were observed at 0.06 g·mL^-1^ (*p<* 0.05), while POD activity was significantly inhibited at 0.10 g·mL^-1^ (*p<* 0.05).

CAT activity in cocozelle seedlings also varied significantly with extract concentration. At 0.06 g·mL^-1^, CAT activity more than doubled compared to the control (*p<* 0.05). However, when the concentration increased to 0.08-0.10 g·mL^-1^, CAT activity decreased significantly, though it remained higher than control level (*p<* 0.05). A similar pattern was observed in corn seedling, but significant enhancement occurred only at 0.08 g·mL^-1^ (*p<* 0.05). At 0.10 g·mL^-1^, CAT activity in corn was significantly lower than the control, with a reduction of up to 25% (*p<* 0.05). In contrast, CAT activity in kidney bean seedlings showed only slight fluctuations across concentrations, with no statistically significant differences among treatments (**Table 5**).

## 4 Discussion

### 4.1 Effects of cabbage-leaf water extract on seed germination and seedling growth of three crops

Numerous studies have shown that plant extracts can significantly inhibit seed germination and seedling growth in other crops. For example, Jiang et al. (2006) reported that the aqueous extract of *Lycoris radiata* L. had a strong inhibitory effect on the seedling growth of radish (*Raphanus sativus* L.), cucumber (*Cucumis sativus* L.), tomato (*Solanum lycopersicum* L.) and rape (*Brassica napus* L.), with greater inhibition at higher concentrations. Similarly, Yu et al. (2013) found that wheat straw extract suppressed seed germination and seedling growth in rice (*Oryza sativa* L.), while Luo et al. (2023) demonstrated that extracts from the root, stem and leaf of chamaejasme (*Euphorbia jolkinii*) inhibited seed germination and seedling growth in perennial ryegrass (*Loium perenne*).

In line with these findings, our study showed that aqueous extracts of cabbage leaves significantly inhibited seed germination and seedling growth in cocozelle, kidney bean and corn. Its inhibition intensity increased with the increase of extract concentration as Li et al. (2022) reported. Across all tested concentrations, the degree of inhibition of germination followed a consistent order: cocozelle > kidney beans > corn. The germination rate of cocozelle seeds was significantly lower than that of kidney bean and corn (*p<* 0.05), indicating that cocozelle was the most sensitive to cabbage leaf allelochemicals (**Figure 1B**). This differential sensitivity may reflect the varying resistance of crop species to the same allelopathic compounds. And similar results have been documented by Ma et al. (2016).

Interestingly, although the extract inhibited radicle elongation in kidney bean, it simultaneously promoted germ biomass accumulation. This suggests that allelochemicals in cabbage leaves may exert contrasting effects on different organs of the same plant, a phenomenon also noted by Jiang et al. (2006).

Generally, allelopathic effects tend to impact radicles more severely than germs, likely because the radicle is the first to encounter allelochemicals during germination. This trend was also observed in studies on *Xanthium sibiricum* allelopathy (Gao et al., 2009), though it contrasts with findings from *Lycoris radiata, which inhibited* germs more strongly than radicles (Jiang et al., 2006). These discrepancies may be attributed to differences in the type and quantity of allelochemicals released by various donor crops (Zuo et al., 2021).

In Shanxi, where our study was conducted, the peak rainfall period coincides with the vegetative growth phase of cabbage, during which secondary metabolite production increases. These compounds can leach into the soil via rain and fog (Zhao et al., 2022). While the soil’s buffering capacity can delay allelopathic effects, high concentrations of allelochemicals can eventually accumulate and impair seedling growth. Based on pre-experimental results, we selected multiple-fold concentrations that inhibited seed germination for use in soil pot trials to simulate field conditions. This method was similar to that used by Luo et al. (2023).

Notably, in early pot trials, 0.01 g·mL^-1^ cabbage leaf extract appeared to promote cocozelle growth, enhancing root elongation, plant height, and aboveground biomass. In the case of corn, concentrations between 0.01-0.03 g·mL^-1^ did not suppress seedling growth development. These results suggest that the allelopathic impact of cabbage may be dose-dependent and crop-specific.

Considering that the intensity of allelopathic effects from water-soluble compounds is influenced by irrigation and precipitation (Jennings and Nelson, 2002), crop selection following cabbage harvest should take rainfall into account. In areas with adequate rainfall, corn is a suitable follow-up crop. In drier regions, kidney bean may be a better choice. This recommendation aligns with findings from Gao et al. (2009), who also observed that different crops show varying sensitivities to *Xanthium sibiricum water extract*.

In conclusion, cabbage-leaf extracts significantly affect seed germination rate, germination potential, germination index, vigor index, seedling length, root length, and biomass accumulation. These effects vary not only among different receptor crops (Jiang et al., 2006) but also within the same crop depending on the treatment concentration (Chen et al., 2024).

### 4.2 Physiological mechanism of allelopathy of cabbage on three crops

When plants are exposed to allelochemicals, the first effect is often damage to the cell membrane. These compounds can alter membrane permeability, disrupt physiological activity and material transport, and ultimately impair plant growth (Peng et al., 2011). MDA, a byproduct of membrane lipid peroxidation, is widely recognized as an important indicator of membrane damage (Yu et al., 2013). As a reactive compound, MDA can interact with proteins and other cellular components, inhibit protein synthesis, damage enzymes and membranes, reduce membrane fluidity and stability, and ultimately compromise membrane integrity. Therefore, MDA content is closely linked to the extent of membrane injury (Tong et al., 2007).

In this study, MDA levels in cocozelle seedlings were slightly lower than the control only under the lowest treatment concentration (0.06 g·mL^-1^), suggesting that cocozelle may regulate MDA accumulation through its antioxidant enzyme system (**Table 5**). This may partly explain the relatively weak allelopathic inhibition observed in cocozelle at low extract concentrations. However, as the concentration increased, MDA content rose accordingly, indicating that once the regulatory threshold was exceeded, damage to the membrane system increased. Similar findings have been reported by Wang et al. (2012) and Gao et al. (2009), although the exact patterns of MDA fluctuation vary depending on the type and concentration of allelochemicals, as well as the receptor crop involved.

Plants under stress also activate a protective mechanism, namely, the antioxidant defense system, to scavenge excess free radicals. This system includes several key enzymes, among which SOD, POD, and CAT play the most important roles. SOD neutralizes superoxide radicals, maintaining cellular redox balance. POD helps prevent lipid peroxidation, reducing membrane damage, while CAT decomposes hydrogen peroxide, another harmful free radical. These enzymes typically work together to mitigate oxidative stress, maintain free radical homeostasis, and protect cell membranes (Gao et al., 2021; Guo et al., 2022).

In this study, antioxidant enzyme activities in cocozelle and corn seedlings increased at moderate concentrations of cabbage-leaf extract, then declined at the highest concentration. Specifically, SOD and POD activities peaked at intermediate concentrations but were significantly lower than the control under maximum stress (**Table 5**). This suggests that low to moderate exposure may activate the antioxidant system, but at high concentrations, radical accumulation overwhelms the plant’s defense capacity, resulting in oxidative damage and elevated membrane lipid peroxidation. These results align with those of Cheng and Cheng (2015), who reported that high concentrations of allelochemicals can disrupt antioxidant defenses. A similar pattern was observed by Yang et al. (2023), who found that sesame water extract compromised the antioxidant enzyme system in *Phyllostachys edulis*, leading to reactive oxygen species buildup and growth inhibition.

In this experiment, light, temperature, and moisture were controlled to eliminate environmental variability, allowing for an objective evaluation of cabbage allelopathy. However, it is important to recognize that under field conditions, the release and impact of allelochemicals are influenced by various factors such as precipitation, climate, soil, plant biomass, litter density, and decomposition rate (Gao et al., 2009). These factors can lead to significant differences between laboratory results and actual field outcomes. Future research should account for soil texture, biological properties, and other release pathways when evaluating the effects of cabbage allelochemicals on crop germination and seedling development.

Using the crop-specific differences in allelopathic response may offer practical solutions. For example, planting species less sensitive to cabbage allelochemicals, like kidney bean, could help alleviate negative stubble effects. Further studies are also needed to identify the specific bioactive compounds, clarify their release pathways, and explore their transformation and application potential in crop rotation systems.

## 5 Conclusion

Allelochemicals released from cabbage leaves, primarily through rain and fog leaching, can negatively impact seed germination and seedling growth in cocozelle, kidney bean, and corn. The overall intensity of allelopathic inhibition followed the order: cocozelle > corn > kidney bean. These differences were closely associated with variations in MDA content and antioxidant enzyme activity in the seedlings. Based on these findings, planting kidney beans after cabbage may be a viable strategy to mitigate allelopathic stress. Additionally, removing the entire aboveground portion of cabbage during harvest is recommended to reduce the accumulation of in the soil and minimize suppression of subsequent crops.

## Data Availability

The original contributions presented in the study are included in the article/**Supplementary Material**. Further inquiries can be directed to the corresponding authors.
