# Supplementary material for: The cabbage-leaf water extract can inhibit the germination and seedling growth of three receptor crops
Source: Front Plant Sci. 2025 Aug 7;16:1609150. doi: 10.3389/fpls.2025.1609150 (PMC12367773; doi:10.3389/fpls.2025.1609150)
Supplement: Supplementary file 1 [file Table1.docx]

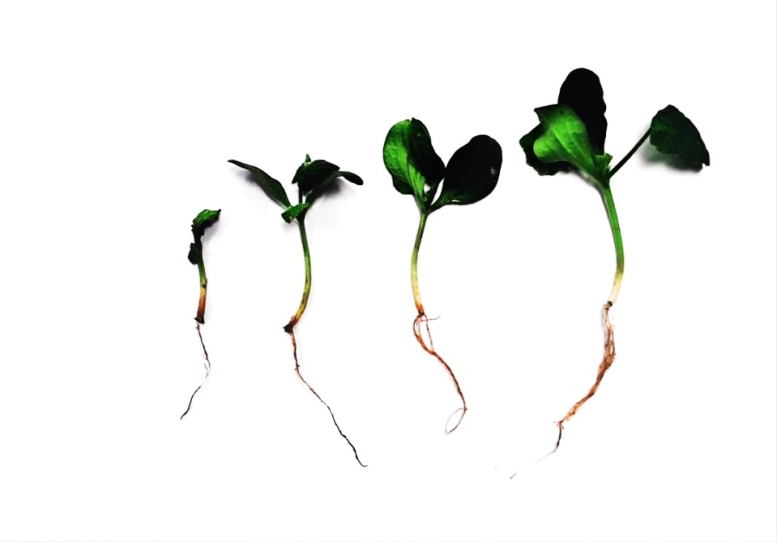


(A)


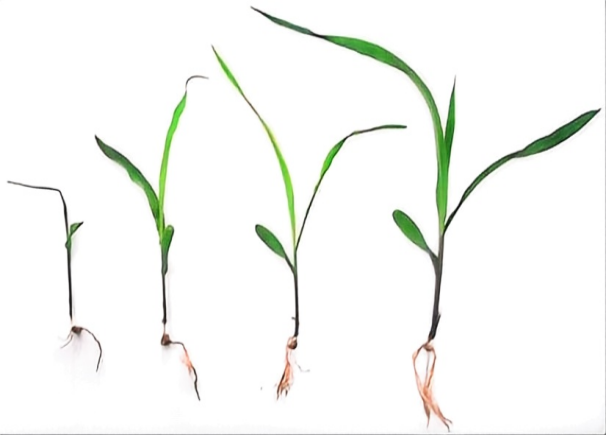


(B)

Figure S1

Cocozelle (A) and corn (B) seedling growth in pot (20d) under different concentrations of cabbage-leaf water extracts. Concentration (g·mL^-1^) from left to right:1: 0.1; 2: 0.08; 3: 0.06; 4: 0 (CK)
